# VIDAS^®^ TB-IGRA reagents induce a CD4+ and CD8+ T-cell IFN-γ response for both TB infection and active TB

**DOI:** 10.5588/ijtld.21.0478

**Published:** 2022-01-01

**Authors:** E. Petruccioli, C. Farroni, G. Cuzzi, V. Vanini, F. Palmieri, P. Vittozzi, D. Goletti

**Affiliations:** 1 Translational Research Unit, National Institute for Infectious Diseases Lazzaro Spallanzani, Istituto di Ricovero e Cura a Carattere Scientifico, Rome, Italy; 2 UOS Professioni Sanitarie Tecniche, National Institute for Infectious Diseases Lazzaro Spallanzani, Istituto di Ricovero e Cura a Carattere Scientifico, Rome, Italy; 3 Respiratory Infectious Diseases Unit, National Institute for Infectious Diseases Lazzaro Spallanzani, Istituto di Ricovero e Cura a Carattere Scientifico, Rome, Italy

Dear Editor,

TB is one of the leading causes of death and the WHO has estimated an additional 9.9 million new TB cases in 2020.^1^ Global control and elimination of TB can be improved by identifying latent TB infection (LTBI) and treating those who are at highest risk of developing active TB.^2^–^5^ Many diagnostic assays are available for LTBI, including the tuberculin skin test (TST) and interferon-gamma (IFN-γ) release assays (IGRAs). QuantiFERON-TB Gold Plus (QTF-Plus; Qiagen, Hilden, Germany) and T-SPOT.*TB* (Oxford Immunotec, Abingdon, UK) are the most routinely used IGRAs.^2^–^4^ The design and development of IGRAs are based on the knowledge that both CD4^+^ and CD8^+^ T-cells are important in TB immune pathogenesis.^6^,^7^ CD4^+^ T-cell response plays an important role, as shown by the high risk of progression to TB disease in subjects with CD4^+^ T-cell impairment, such as HIV-infected individuals.^1^ CD8-specific T-cells are involved in the control of *Mycobacterium tuberculosis* (MTB) bacterial load, and are increased at TB disease diagnosis and reduced at the end of therapy.^8^,^9^ A new assay has recently been developed (VIDAS^®^ TBIGRA, bioMérieux, Marcy-l’Étoile, France) based on early secretory antigenic target-6 and culture filtrate protein-10 peptides^10^ for the diagnosis of individuals infected with MTB, both disease and infection. In a recent multicentre study, this test showed a higher accuracy for active TB than QFTPlus, used as surrogate for TB infection, with 97.5% sensitivity.^11^,^12^ The assay is completely automated; the collected whole blood is dispensed from a single tube into three different wells: NIL (negative control), AG (MTB antigen) and Mitogen (positive control). After on-board incubation, IFN-γ is measured using VIDAS 3 instrument.^12^ Here, we aimed to characterise the T-cell-specific response induced by VIDAS TB-IGRA stimulation reagents using flow cytometry to analyse the CD4^+^ and CD8^+^ T-cell response in relation to a subject’s TB status.

**Figure 1:**
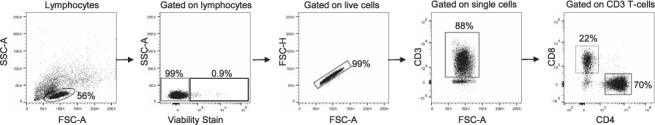
Gating strategy for IFN-γ analysis in T-cell subsets and IFN-γ production by CD4^+^ and CD8^+^ T-cells in patients with active TB and LTBI. Plots show the distribution and frequency of IFN-γ in PBMCs of a representative active TB and LTBI subject. Lymphocytes were gated based on their SSC-A and FSC-A parameters, following which only live cells were considered according to the negativity of the Viability Stain antibody and single cells were gated as described. CD3^+^ T-cells were gated according to the expression of their surface marker. SSC-A = side scatter; FSC-A = forward scatter; IFN-γ = interferon-gamma; LTBI = latent TB infection; PBMC = peripheral blood mononuclear cell.

Between November 2020 and January 2021, peripheral blood mononuclear cells (PBMCs) were taken from patients with active TB and LTBI. Active TB patients were enrolled within 7 days from starting treatment, and those with LTBI before starting preventive treatment. Active TB was microbiologically confirmed using culture testing and/or nucleic acid tests. All enrolled individuals were tested using QTF-Plus assay. PBMCs were isolated, frozen and stored in liquid nitrogen until use. For flow cytometry assay, 1×10^6^ PBMCs were stimulated with VIDAS TB-IGRA reagents (bioMérieux): NIL (the inert compound contained in the buffer employed to dilute the antigen peptides that was used as negative control, 10 μg/ml), AG (MTB antigen, 100 μg/ml), Mitogen (positive control, 20 μg/ml). Anti-CD28 and anti-CD49d (1 μg/ml each) monoclonal antibodies and Golgi Plug™ (1 μg/ml) were added to PBMCs, as previously described.^7^ After 16–24 h, PBMCs were stained using CD3 PE-Cy7, CD4 BV711 and CD8 APC-H7. Cytofix/Cytoperm™ was used for IFN-γ-APC intracellular staining according to the manufacturer’s recommendations (Figure 1) (all antibodies and reagents from BD Biosciences, San Jose, CA, USA). Samples were acquired using a BD Lyric cytometer (BD Biosciences). Dead cells were excluded by both side/forward scatter using BD Horizon™ Fixable Viability Stain 700 (BD Bioscience). Data were analysed using FlowJo software v10 (Tree Star, Ashland, OR, USA). Cytokine background was subtracted from the stimulated tubes. Detection of at least 10 events in the CD4^+^ IFN-γ^+^ or CD8^+^ IFN-γ^+^ gate was considered a positive response.^14^ Statistical analysis was performed using Prism software (Graph-Pad Prism 8, La Jolla, CA, USA). Median and interquartile ranges (IQRs) were calculated for continuous measures, χ^2^ test for proportions, and Mann-Whitney *U*-test for pairwise comparison. Data were considered statistically significant when *P* < 0.05. All participants provided written informed consent; the study was approved by the Ethical Committee of Istituto Nazionale Malattie Infettive Lazzaro Spallanzani-IRCCS, Rome, Italy (approval 126/2020 and 58/2018) and complied with the principles of the Declaration of Helsinki.^13^

**Figure 2:**
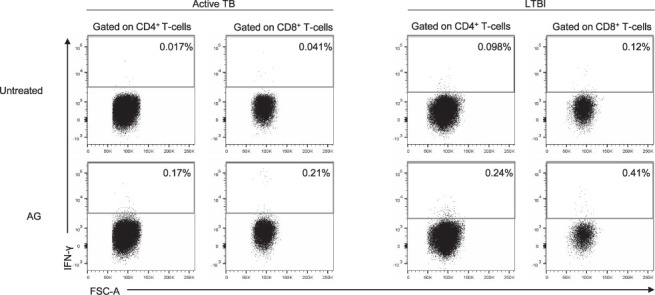
Percentage of IFN-γ^+^ CD4^+^ T-cells and IFN-γ^+^ CD8^+^ T-cells in untreated cells and in response to AG stimulation in LTBI subjects and active TB patients. LTBI = latent TB infection; AG = MTB antigen; IFN-γ = interferon-gamma; FSC-A = forward scatter.

**Figure 3:**
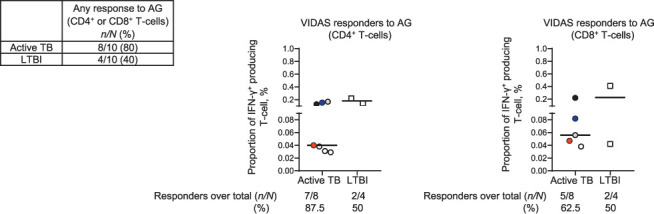
Left: Box showing both numbers and percentage of participants who showed a CD4^+^ or/and CD8^+^ T-cell response. Middle and right graphs: percentage of CD4^+^, CD8^+^/IFN-γ^+^ T-cells in the responders to VIDAS TB-IGRA AG. Each dot/square represents a single active TB or LTBI patient, black lines represent median values. Individuals who showed both CD4^+^ and CD8^+^ responses are represented with the same colour in all graphs. Individuals who showed a single CD4^+^ or CD8^+^ T-cell response are indicated in white. No differences between groups were observed using the Mann–Whitney test. AG = (VIDAS TB-IGRA) antigen tube reagent; LTBI = latent TB infection; IFN-γ = interferon-gamma.

We enrolled 10 LTBI and 10 active TB individuals. Among the LTBI subjects, 9 were recent contacts of pulmonary TB patients and 1 was a remote infection.^2^ We did not find significant differences for age (active TB: median age 33 years, IQR 24–49; LTBI: median age 44 years, IQR 28–52), sex (active TB: female 20%, LTBI: female 30%), bacilli Calmette-Guérin vaccination (active TB: 60%; LTBI: 60%) and origin (West Europe active TB: 40%, LTBI: 40%; East Europe active TB: 60%; LTBI: 50%) between the two groups. No significant differences in QTF-Plus scores between active TB and LTBI were reported (active TB: 70%, LTBI: 100%). We performed an intracellular staining to evaluate the frequency of CD4^+^ IFN-γ^+^ and CD8^+^ IFN-γ^+^ T-cells (Figure 2). Regardless of the CD4^+^ or CD8^+^ T-cell subset involved, we scored 8/10 active TB responders (80%), and 4/10 LTBI responders (40%) (Figure 3). In particular, 87.5% of the active TB patients and 50% of the LTBI subjects had a CD4^+^ T-cell-positive response to the AG (Figure 3). Similarly, 62.5% of active TB and 50% of LTBI samples showed a CD8^+^ T-cell response (Figure 3). Although we did not find any significant difference, the frequency of CD4^+^ IFN-γ^+^ and CD8^+^ IFN-γ^+^ T-cells was higher in LTBI than in active TB. All samples scored positive for the mitogen-control, indicating that participants were immunocompetent (data not shown), and had a cell viability between 89.4% and 99.7% (data not shown).

Here, for the first time, using a flow cytometry approach we have demonstrated that the AG reagent of VIDAS TB-IGRA induces both CD4^+^ and CD8^+^ T-cell responses in a cohort of subjects with active TB and LTBI enrolled in a low TB endemic country such as Italy. Previous studies have reported the ability of other IGRAs, such as the QFT-Plus, to induce both a CD4^+^ and CD8^+^ T-cell response in active TB and LTBI subjects with or without HIV-infection.^2^,^3^ In the present study, we confirmed this by using a different test. We also observed a higher number of CD8^+^ T-cell responses to VIDAS TBIGRA AG in active TB than in LTBI; this has been previously shown using the QFT-Plus assay in both HIV-uninfected and -infected subjects.^3^,^8^ Interestingly, we also found CD8^+^ T-cell responders in LTBI. This is due to the fact that TB comprises a spectrum of different stages characterised by host-pathogen equilibrium;^15^ it is therefore not uncommon to detect a CD8^+^ T-cell response during LTBI as well. In this study, only 4/10 of the enrolled LTBI subjects scored positive on VIDAS TB-IGRA AG using flow cytometry, although all LTBI subjects enrolled in our study had a positive response to the QFT-Plus test. This is likely due to the use of frozen PBMCs that may limit the detection of specific responses compared to fresh samples.^16^ In conclusion, we have shown that VIDAS TB-IGRA reagents induce both CD4^+^ and CD8^+^ T-cell IFN-γ response in both LTBI and active TB patients. Our findings provide further details on the immunological features of the responses associated with the test and on the contribution of T-cell subpopulations. These results are important in shedding light on VIDAS TB-IGRA characteristics, a new generation of completely automated IGRA.^12^
